# Chaos Control and Synchronization of a Complex Rikitake Dynamo Model

**DOI:** 10.3390/e22060671

**Published:** 2020-06-17

**Authors:** Wenkai Pang, Zekang Wu, Yu Xiao, Cuimei Jiang

**Affiliations:** School of Mathematics and Statistics, Qilu University of Technology (Shandong Academy of Sciences), Jinan 250353, China; p_wenkai@163.com (W.P.); Wu_Yulun8@163.com (Z.W.); 18365793561@163.com (Y.X.)

**Keywords:** complex Rikitake system, chaos control, existence, coexistence, synchronization, adaptive feedback control, 34C28, 34D06

## Abstract

A novel chaotic system called complex Rikitake system is proposed. Dynamical properties, including symmetry, dissipation, stability of equilibria, Lyapunov exponents and bifurcation, are analyzed on the basis of theoretical analysis and numerical simulation. Further, based on feedback control method, the complex Rikitake system can be controlled to any equilibrium points. Additionally, this paper not only proves the existence of two types of synchronization schemes in the complex Rikitake system but also designs adaptive controllers to realize them. The proposed results are verified by numerical simulations.

## 1. Introduction

Since the pioneer research work of Ott et al. [1], Pecora and Carroll [2], the topic of chaos control and synchronization has attracted a lot of researchers in diverse areas including mathematics, physics, biology, medicine, engineering, and so on. Lots of research has been paid to study chaos control for real systems, and plenty of control methods have been put forward, such as feedback control [3,4], sliding mode control [5,6], backstepping method [7], and so on. These control strategies can also be employed to realize various kinds of synchronization of real chaos. Further developments in this direction can be found in [8,9,10,11,12,13,14].

The quoted literature above are only related to real chaotic systems and do not consider the chaotic systems which consist of complex variables. As is known to all, in the real world, many cases exist in the form of complex variables. For instance, Fowler et al. [15] discovered the complex Lorenz system when they studied laser physics and baroclinic instability of the geophysical flows in 1982. Since then, the study on complex nonlinear systems has been paid a substantial amount of attentions and has become a hot topic due to its wide applications in chemical systems, optics and especially in secure communications [16,17,18]. A considerable amount of complex dynamical systems exhibit chaotic motion, such as the complex Chen system [19], the time-delay complex Lorenz system [20], the complex generalised Lorenz hyperchaotic system [21], just to name a few examples. Compared with real chaos, complex chaos has the diversity of synchronization types and results. On the one hand, a lot of authors extend some synchronization schemes of real chaos into complex space, for example, complete synchronization (CS) [22], anti-synchronization (AS) [23], lag synchronization (LS) [24], combination synchronization [25], etc. On the other hand, some new synchronization schemes have been proposed on the basis of the characteristics of complex systems, such as complex complete synchronization (CCS) [26], complex lag synchronization (CLS) [27], complex anti lag synchronization (CALS) [28], combination complex synchronization [29,30], and so forth. However, the existing results on complex chaos have three disadvantages: Firstly, chaos control of the complex dynamical systems has gained little attention. Secondly, the existence of the synchronization problem, which is fundamental theoretical base, has not been considered so far. Finally, most of the current designed controllers eliminate the nonlinear term of the system, which are not only complicated but also difficult to realize in engineering. Therefore, control and synchronization in complex chaotic systems needs to be further and extensively studied.

Motivated by the aforementioned discussion, the current investigation concentrates on chaos control and synchronization of a novel complex dynamical system named as complex Rikitake system, which is proposed based on the Rikitake system. Following the idea of studying dynamics in chaotic systems, this paper investigates symmetry, dissipation, stability of equilibria, Lyapunov exponents, Poincaré-sections and bifurcation of the complex Rikitake system. Thus, along with the deeper understanding of feedback control method presented in [9], we construct simple adaptive controllers to realize control and synchronization of the complex Rikitake system. Furthermore, we obtain a criterion to detect the existence of synchronization in the complex Rikitake system and further prove that there exist CS and the coexistence of CS and AS.

The main construct of the article is arranged as follows. We present the complex Rikitake system and analyze some basic dynamics in Section 2. In Section 3, adaptive controllers are designed to control the complex Rikitake system to any equilibrium points. Section 4 gives the main results on chaos synchronization of the complex Rikitake system. The conclusions are provided in Section 5.

## 2. A Complex Chaotic Rikitake Dynamo System

**Figure 1:**
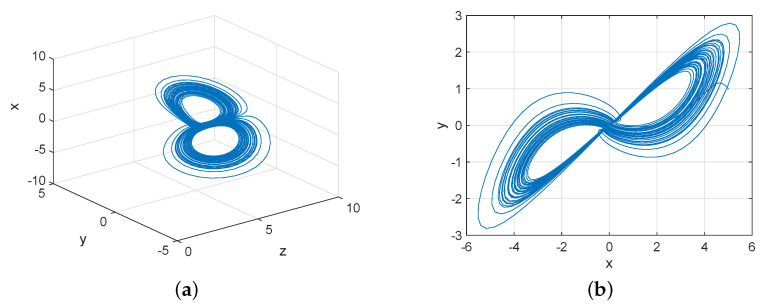
The projection of chaotic attractor for the Rikitake dynamo system (1). (**a**) in the z-y-x space; (**b**) in x-y space.

In 1958, Rikitake discovered the 3-D Rikitake dynamo system [31] whose equations are

(1) $$\begin{array}{c} \begin{array}{c} \left\{\begin{array}{c} \dot{x}=-\beta x+yz,\\ \dot{y}=-\beta y-\alpha x+xz,\\ \dot{z}=1-xy, \end{array}\right. \end{array} \end{array}$$

where x,y,z∈R are state variables, α,β>0 are parameters. As mentioned in [32], the Rikitake system (1) behaves chaotically for α=5 and β=2 with $\left(x_{0},y_{0},z_{0}\right)=\left(3,1,6\right)$, which are shown in Figure 1.

A new system can be generated by assuming that *x* and *y* are complex states and changing cross coupled terms *x* and *y* to complex conjugate form. Thus, we call it complex Rikitake system, which can be described as

(2) $$\begin{array}{c} \begin{array}{c} \left\{\begin{array}{c} \dot{x}=-\beta x+yz,\\ \dot{y}=-\beta y-\alpha x+xz,\\ \dot{z}=1-\frac{1}{2}\left(\bar{x}y+x\bar{y}\right), \end{array}\right. \end{array} \end{array}$$

where $x=x_{1}+jx_{2}$, $y=x_{3}+jx_{4}$, $z=x_{5}$, j=−1, $\bar{x}$ and $\bar{y}$ denote the complex conjugates of *x* and *y*. Replacing x,y in system (2) with real and imaginary variables can lead to the following equivalent system

(3) $$\begin{array}{c} \begin{array}{c} \left\{\begin{array}{c} {\dot{x}}_{1}=-\beta x_{1}+x_{3}x_{5},\\ {\dot{x}}_{2}=-\beta x_{2}+x_{4}x_{5},\\ {\dot{x}}_{3}=-\beta x_{3}-\alpha x_{1}+x_{1}x_{5},\\ {\dot{x}}_{4}=-\beta x_{4}-\alpha x_{2}+x_{2}x_{5},\\ {\dot{x}}_{5}=1-x_{1}x_{3}-x_{2}x_{4}. \end{array}\right. \end{array} \end{array}$$

In the next subsection, we study some dynamical properties of this new system (3).

### 2.1. Symmetry

Given a coordinate transformation *T* as follows

$$T\left(x_{1},x_{2},x_{3},x_{4},x_{5}\right)⟶\left(-x_{1},-x_{2},-x_{3},-x_{4},x_{5}\right).$$

It is clear that each trajectory is symmetrical with respect to the $x_{5}$-axis. That means system (3) is invariant for the given transformation *T*.

### 2.2. Dissipation

The divergence of system (3) can be calculated as

$$\begin{array}{c} \nabla V=\sum_{l=1}^{5}\frac{\partial {\dot{x}}_{l}}{\partial x_{l}}=-4\beta . \end{array}$$

As a result, it follows from the condition β>0 that system (3) is dissipative.

### 2.3. Equilibria and Stability

In order to find the equilibria of system (3), we consider equations in the form

$$\begin{array}{c} \begin{array}{c} \left\{\begin{array}{c} -\beta x_{1}+x_{3}x_{5}=0,\\ -\beta x_{2}+x_{4}x_{5}=0,\\ -\beta x_{3}-\alpha x_{1}+x_{1}x_{5}=0,\\ -\beta x_{4}-\alpha x_{2}+x_{2}x_{5}=0,\\ 1-x_{1}x_{3}-x_{2}x_{4}=0. \end{array}\right. \end{array} \end{array}$$

After computation, we obtain the following equilibrium points:

$$S=(\frac{cos\theta }{r},\frac{sin\theta }{r},rcos\theta ,rsin\theta ,\frac{\alpha +\sqrt{\alpha^{2}+4\beta^{2}}}{2}),$$

where $r=\sqrt{\frac{2\beta }{\alpha +\sqrt{\alpha^{2}+4\beta^{2}}}}$ and θ∈[0,2π]. Now, we consider the stability of *S*. The Jacobian of system (3) at point *S* is deduced as:

$$\begin{array}{c} J_{S}=\left(\begin{array}{ccccc} -\beta & 0 & \frac{\alpha +\sqrt{\alpha^{2}+4\beta^{2}}}{2} & 0 & rcos\theta \\ 0 & -\beta & 0 & \frac{\alpha +\sqrt{\alpha^{2}+4\beta^{2}}}{2} & rsin\theta \\ \frac{\sqrt{\alpha^{2}+4\beta^{2}}-\alpha }{2} & 0 & -\beta & 0 & \frac{cos\theta }{r}\\ 0 & \frac{\sqrt{\alpha^{2}+4\beta^{2}}-\alpha }{2} & 0 & -\beta & \frac{sin\theta }{r}\\ -rcos\theta & -rsin\theta & -\frac{cos\theta }{r} & -\frac{sin\theta }{r} & 0 \end{array}\right). \end{array}$$

Furthermore, one can get the characteristic polynomial of $J_{S}$,

$$\begin{array}{c} \lambda \left(\lambda +2\beta \right)(\lambda^{3}+2\beta \lambda^{2}+\left(\frac{4\beta }{\alpha +\sqrt{\alpha^{2}+4\beta^{2}}}+\frac{\alpha }{\beta }\right)\lambda +\alpha +\sqrt{\alpha^{2}+4\beta^{2}}+\frac{4\beta^{2}}{\alpha +\sqrt{\alpha^{2}+4\beta^{2}}})=0. \end{array}$$

According to Routh–Hurwitz criterion, it is unstable for any given α>0 and β>0.

### 2.4. Chaotic Behavior and Attractors

**Figure 2:**
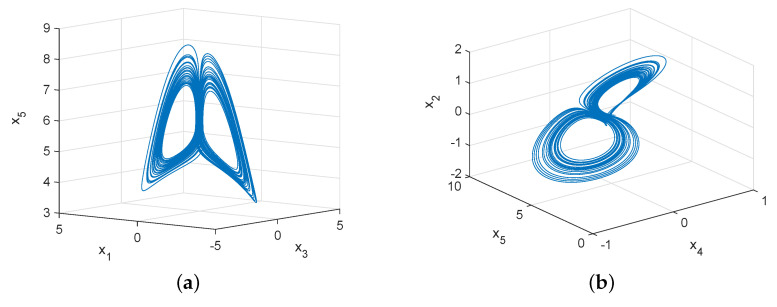

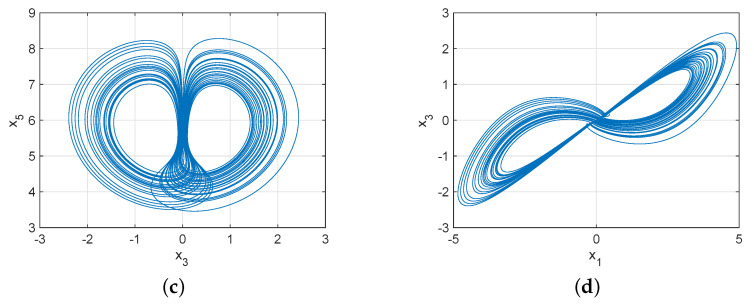
Chaotic attractors of system (3) in different spaces. (**a**) in $x_{3}-x_{1}-x_{5}$ space; (**b**) in $x_{4}-x_{5}-x_{2}$ space; (**c**) in $x_{3}-x_{5}$ space; (**d**) in $x_{1}-x_{3}$ space.

**Figure 3:**
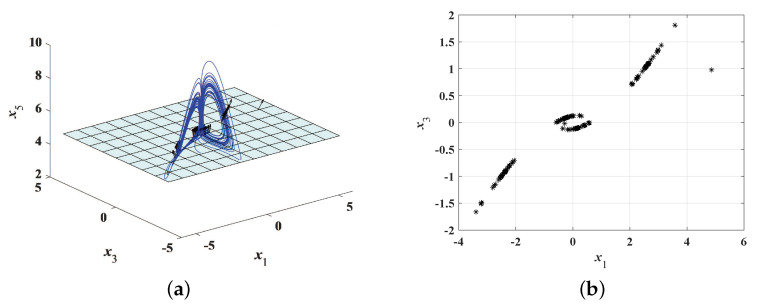
Poincaré map of system (3) with α=5 and β=2. (**a**) in $x_{1}-x_{3}-x_{5}$ space; (**b**) in $x_{1}-x_{3}$ space.

**Figure 4:**
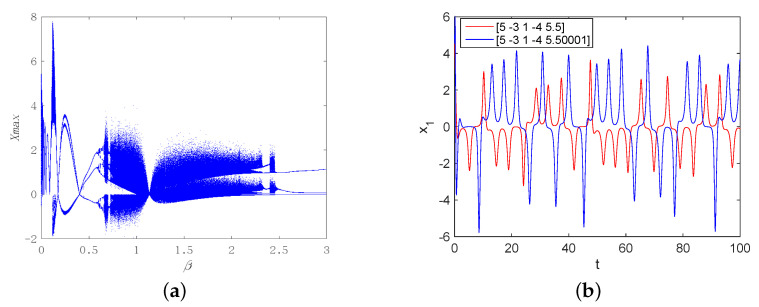
(**a**) Bifurcation diagram of system (3) with α=5; (**b**) State variable under different initial values.

Assuming that α=5, β=2 and $x\left(0\right)={\left(5-3j,1-4j,5.5\right)}^{T}$, the methods numerical analysis are used to obtain chaotic attractor, Poincaré map and bifurcation diagrams, see Figure 2, Figure 3 and Figure 4. Figure 2 shows chaotic attractors of the complex Rikitake system in different planes. The Poincaré diagrams of system (3) are depicted in Figure 3. As described in Figure 4a, basic bifurcation versus parameter β∈(0,3) with α=5. Figure 4b demonstrates system (3) is sensitivity to initial value. Furthermore, we apply numerical computation to obtain the corresponding Lyapunov exponents of system (3),

$$LE_{1}=0.117534,LE_{2}=-0.043679,LE_{3}=-0.051743,LE_{4}=-3.957388,\mathrm{and}\;LE_{5}=-4.064725.$$

Thus, using the formula of fractal dimension [33], we easily deduce that

$$D=j+\frac{1}{|LE_{j+1}|}\sum_{i=1}^{j}LE_{i}=3+\frac{LE_{1}+LE_{2}+LE_{3}}{|LE_{4}|}=3.0055875.$$

This implies that the Lyapunov dimension of system (3) is fractional. Consequently, it is noticeable that system (3) behaves chaotically for this choice of α=5 and β=2.

## 3. Chaos Control

Adaptive technique is adopted to investigate the control problem of the complex Rikitake system. Before giving the conclusion of this section, we first introduce a lemma.

**Lemma** **1** ([9]). *Consider the nonlinear system*

(4) $$\begin{array}{c} \dot{\vartheta }=\varphi \left(\vartheta \right), \end{array}$$

*where $\vartheta \in R^{n}$ is the state, $\varphi \left(\vartheta \right)\in R^{n}$ is continuous function with φ(0)=0. Suppose that there exists a nonsingular coordinate transformation υ=Tϑ, which can convert system (4) into two subsystems*

$$\begin{array}{c} \begin{array}{c} {\dot{\mu }}_{1}=G_{1}\left(\mu_{1},\mu_{2}\right),\\ {\dot{\mu }}_{2}=G_{2}\left(\mu_{1},\mu_{2}\right), \end{array} \end{array}$$

*where $\mu_{1}={\left(\upsilon_{1},\upsilon_{2},\ldots ,\upsilon_{r}\right)}^{T},r\geq 1$, $\mu_{2}={\left(\upsilon_{r+1},\upsilon_{r+2},\ldots ,\upsilon_{n}\right)}^{T}$, $G_{1}\left(\mu_{1},\mu_{2}\right)\in R^{r}$, $G_{2}\left(\mu_{1},\mu_{2}\right)\in R^{n-r}$, and the subsystem*

$$\begin{array}{c} {\dot{\mu }}_{2}=G_{2}\left(0,\mu_{2}\right) \end{array}$$

*is globally asymptotically stable (GAS). Then the controller is designed as*

$$\begin{array}{c} u={\left(k\mu_{1},0\right)}^{T} \end{array}$$

*and the adaptation law is in the form of*

$$\begin{array}{c} \dot{k}=-\sigma \mu_{1}^{T}\mu_{1}, \end{array}$$

*where σ>0 is an arbitrary real number. That is to say, the controlled system*

$$\begin{array}{c} \begin{array}{c} {\dot{\mu }}_{1}=G_{1}\left(\mu_{1},\mu_{2}\right)+k\mu_{1},\\ {\dot{\mu }}_{2}=G_{2}\left(\mu_{1},\mu_{2}\right) \end{array} \end{array}$$

*is asymptotically stable.*

As discussed in Section 2, the complex Rikitake system has no stable equilibrium point. Next, we design a feedback controller to stabilize the complex Rikitake system to any fixed points. The equilibrium point of system (3) is recorded as $S=(s_{1},s_{2},s_{3},s_{4},s_{5})$. Making the following coordinate transformation:

$$\begin{array}{c} \begin{array}{c} \left\{\begin{array}{c} x_{1}={\tilde{x}}_{1}+s_{1},\\ x_{2}={\tilde{x}}_{2}+s_{2},\\ x_{3}={\tilde{x}}_{3}+s_{3},\\ x_{4}={\tilde{x}}_{4}+s_{4},\\ x_{5}={\tilde{x}}_{5}+s_{5}, \end{array}\right. \end{array} \end{array}$$

we further have the controlled system

(5) $$\begin{array}{c} \begin{array}{c} \left\{\begin{array}{c} {\dot{\tilde{x}}}_{1}=-\beta {\tilde{x}}_{1}-{\tilde{x}}_{3}{\tilde{x}}_{5}+{\tilde{x}}_{3}s_{5}+{\tilde{x}}_{5}s_{3}+u_{1},\\ {\dot{\tilde{x}}}_{2}=-\beta {\tilde{x}}_{2}-{\tilde{x}}_{4}{\tilde{x}}_{5}+{\tilde{x}}_{4}s_{5}+{\tilde{x}}_{5}s_{4}+u_{2},\\ {\dot{\tilde{x}}}_{3}=-\beta {\tilde{x}}_{3}+{\tilde{x}}_{1}{\tilde{x}}_{5}+{\tilde{x}}_{1}s_{5}+{\tilde{x}}_{5}s_{1}-\alpha {\tilde{x}}_{1}+u_{3},\\ {\dot{\tilde{x}}}_{4}=-\beta {\tilde{x}}_{4}+{\tilde{x}}_{2}{\tilde{x}}_{5}+{\tilde{x}}_{2}s_{5}+{\tilde{x}}_{5}s_{2}-\alpha {\tilde{x}}_{2}+u_{4},\\ {\dot{\tilde{x}}}_{5}=-{\tilde{x}}_{1}{\tilde{x}}_{3}-{\tilde{x}}_{1}s_{3}-{\tilde{x}}_{3}s_{1}-{\tilde{x}}_{2}{\tilde{x}}_{4}-{\tilde{x}}_{2}s_{4}-{\tilde{x}}_{4}s_{2}+u_{5}, \end{array}\right. \end{array} \end{array}$$

where $u={\left(u_{1},u_{2},u_{3},u_{4},u_{5}\right)}^{T}$ is the controller to be designed. Thus, the problem of stabilizing system (3) to the equilibrium point *S* is converted to that of stabilizing system (5) at the origin. By Lemma 1, we have the following result.

**Theorem** **1.** *System (3) can be controlled to the equilibrium point $S=(s_{1},s_{2},s_{3},s_{4},s_{5})$ by constructing the following adaptive feedback controller*

(6) $$\begin{array}{c} \begin{array}{c} \left\{\begin{array}{c} u={\left(k{\tilde{x}}_{1},k{\tilde{x}}_{2},0,0,0\right)}^{T},\\ \dot{k}=-\sigma \left({\tilde{x}}_{1}^{2}+{\tilde{x}}_{2}^{2}\right), \end{array}\right. \end{array} \end{array}$$

*where σ>0 is a chosen positive real number.*

**Proof.**  It is noticeable that when ${\tilde{x}}_{1}={\tilde{x}}_{2}=0$, the remainder subsystem of system (5) without a controller becomes

(7) $$\begin{array}{c} \begin{array}{c} \left\{\begin{array}{c} {\dot{\tilde{x}}}_{3}=-\beta {\tilde{x}}_{3}+{\tilde{x}}_{5}s_{1},\\ {\dot{\tilde{x}}}_{4}=-\beta {\tilde{x}}_{4}+{\tilde{x}}_{5}s_{2},\\ {\dot{\tilde{x}}}_{5}=-{\tilde{x}}_{3}s_{1}-{\tilde{x}}_{4}s_{2}. \end{array}\right. \end{array} \end{array}$$

The coefficient matrix of system (7) is

$$\begin{array}{c} J=\left(\begin{array}{ccc} -\beta & 0 & s_{1}\\ 0 & -\beta & s_{2}\\ -s_{1} & -s_{2} & 0 \end{array}\right), \end{array}$$

and its corresponding characteristic equation is described by

(8) $$\begin{array}{c} \left(\lambda +\beta \right)\left(\lambda^{2}+\beta \lambda +s_{1}^{2}+s_{2}^{2}\right)=0. \end{array}$$

Through the analysis of Equation (8), we conclude all roots have negative real part. Thus, according to Routh–Hurwitz criterion, system (7) is GAS. By Lemma 1, one deduces that system (5) can be regulated at the origin by controller (6), that is, system (3) tends to the equilibrium point *S*. □

By the same argument, when ${\tilde{x}}_{3}={\tilde{x}}_{4}=0$, the subsystem of system (5) without controller is of the form

$$\begin{array}{c} \begin{array}{c} \left\{\begin{array}{c} {\dot{\tilde{x}}}_{1}=-\beta {\tilde{x}}_{1}+{\tilde{x}}_{5}s_{3},\\ {\dot{\tilde{x}}}_{2}=-\beta {\tilde{x}}_{2}+{\tilde{x}}_{5}s_{4},\\ {\dot{\tilde{x}}}_{5}=-{\tilde{x}}_{1}s_{3}-{\tilde{x}}_{2}s_{4}, \end{array}\right. \end{array} \end{array}$$

which is GAS. We derive another result on stabilization of the complex Rikitake system from Lemma 1.

**Theorem** **2.** *System (3) can be regulated to the equilibrium point $S=(s_{1},s_{2},s_{3},s_{4},s_{5})$ by constructing the following adaptive feedback controller*

(9) $$\begin{array}{c} \begin{array}{c} \left\{\begin{array}{c} u={\left(0,0,k{\tilde{x}}_{3},k{\tilde{x}}_{4},0\right)}^{T},\\ \dot{k}=-\sigma \left({\tilde{x}}_{3}^{2}+{\tilde{x}}_{4}^{2}\right), \end{array}\right. \end{array} \end{array}$$

*where σ>0 is an arbitrary real number.*

Based on the proposed results, we will now give a numerical description on controlling the complex Rikitake system. In the following two cases, choose the parameters as α=5, β=2, and fix the initial values as $x\left(0\right)={\left(5-3j,1-4j,5.5\right)}^{T}$.

**Figure 5:**
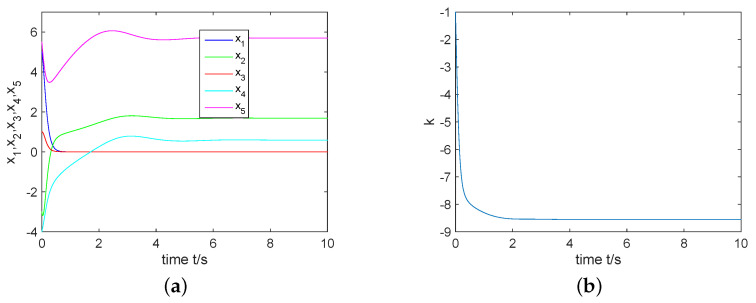
(**a**) Control the complex Rikitake system (3) to $S_{1}$; (**b**) *k* tends to a negative constant.

For the choice of $\theta =\frac{\pi }{2}$, an unstable equilibrium point can be obtained as $S_{1}=\left(0,1.689,0,0.5923,5.702\right)$. From Theorem 1, we derive the controller (6) with σ(0)=1 and k(0)=−1. From Figure 5, one can see that the complex Rikitake system (3) can be regulated to its equilibrium point $S_{1}$ via the controller (6).

**Figure 6:**
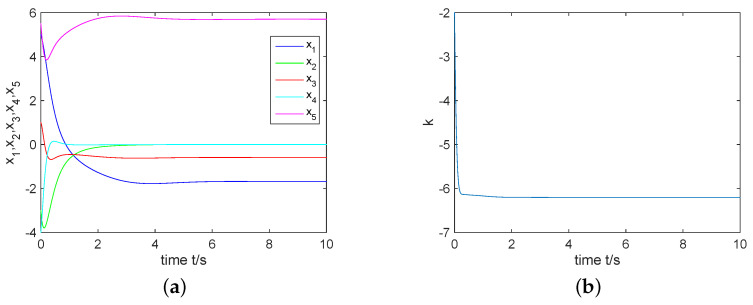
(**a**) Control the complex Rikitake system (3) to $S_{2}$; (**b**) *k* approaches to a negative constant.

For the choice of θ=0, an unstable equilibrium point can be obtained as $S_{2}=\left(-1.689,0,-0.5923,0,5.702\right)$. From Theorem 2, we derive the controller (9) with σ(0)=3 and k(0)=−2. As shown in Figure 6, the complex Rikitake system (3) converges to its equilibrium point $S_{2}$.

## 4. Synchronization Scheme

This section proves the existence of synchronization of the complex Rikitake system, and then realizes CS and the coexistence of CS and AS by feedback control method.

Let us consider two identical complex Rikitake systems with different initial conditions. The drive system is described by

(10) $$\begin{array}{c} \begin{array}{c} \dot{Y}=h\left(Y\right), \end{array} \end{array}$$

where $Y={\left(y_{1},y_{2},y_{3}\right)}^{T}\in C^{3}$, $y_{1}=y_{1}^{r}+jy_{1}^{i}$, $y_{2}=y_{2}^{r}+jy_{2}^{i}$, $h\left(Y\right)={\left(h_{1}\left(Y\right),h_{2}\left(Y\right),h_{3}\left(Y\right)\right)}^{T}\in C^{3}$, $y_{3}\in R$, and

$$\begin{array}{c} \begin{array}{c} h_{1}\left(Y\right)=-\beta y_{1}+y_{2}y_{3},\\ h_{2}\left(Y\right)=-\beta y_{2}-\alpha y_{1}+y_{1}y_{3},\\ h_{3}\left(Y\right)=1-\frac{1}{2}\left({\bar{y}}_{1}y_{2}+y_{1}{\bar{y}}_{2}\right). \end{array} \end{array}$$

In the same way, the response system with controllers can be expressed as

(11) $$\begin{array}{c} \begin{array}{c} \dot{Z}=h\left(Z\right)+u, \end{array} \end{array}$$

where $Z={\left(z_{1},z_{2},z_{3}\right)}^{T}\in C^{3}$, $z_{1}=z_{1}^{r}+jz_{1}^{i}$, $z_{2}=z_{2}^{r}+jz_{2}^{i}$, $u={\left(u_{1},u_{2},u_{3}\right)}^{T}\in C^{3}$ is the error feedback controller to be designed, $z_{3}\in R$, and

$$\begin{array}{c} \begin{array}{c} h_{1}\left(Z\right)=-\beta z_{1}+z_{2}z_{3},\\ h_{2}\left(Z\right)=-\beta z_{2}-\alpha z_{1}+z_{1}z_{3},\\ h_{3}\left(Z\right)=1-\frac{1}{2}\left({\bar{z}}_{1}z_{2}+z_{1}{\bar{z}}_{2}\right). \end{array} \end{array}$$

The synchronization error is denoted as

$$\begin{array}{c} \begin{array}{c} e=Z-\delta Y, \end{array} \end{array}$$

where $\delta =diag\{\delta_{1},\delta_{2},\delta_{3}\}$ and $\delta_{i}\neq 0$ are real constants (i=1,2,3).

Following the results in [12], we introduce the relevant definition.

**Definition** **1.** *For the drive system (10) and the response system (11),*

*1.*  *Systems (10) and (11) are said to be CS if there exists a diagonal matrix $\delta =I_{3},$ i.e., $\delta_{i}=1$ (i=1,2,3), such that $\lim_{t\to \infty }\left||e\left(t\right)|\right|=0$;*
*2.*  *Systems (10) and (11) are said to be AS if there exists a diagonal matrix $\delta =-I_{3},$ i.e., $\delta_{i}=-1$ (i=1,2,3), such that $\lim_{t\to \infty }\left||e\left(t\right)|\right|=0$;*
*3.*  *Systems (10) and (11) are said to achieve the coexistence of CS and AS if there exist some $\delta_{i}=1$ while the remaining $\delta_{j}=-1$ (i≠j, i,j=1,2,3), such that $\lim_{t\to \infty }\left||e\left(t\right)|\right|=0$.*

### 4.1. The Existence of Synchronization in the Complex Rikitake System

Taking the derivative of e(t) and using Equations (10) and (11), one obtains

$$\begin{array}{c} \begin{array}{c} \dot{e}=h\left(Z\right)-\delta h\left(Y\right)+u, \end{array} \end{array}$$

which is equivalent to the following equations

$$\begin{array}{c} \begin{array}{c} {\dot{e}}^{r}=h^{r}\left(Z\right)-\delta h^{r}\left(Y\right)+u^{r}, \end{array} \end{array}$$

and

$$\begin{array}{c} \begin{array}{c} {\dot{e}}^{i}=h^{i}\left(Z\right)-\delta h^{i}\left(Y\right)+u^{i}. \end{array} \end{array}$$

It is clear that e=0 implies $e^{r}=0$ and $e^{i}=0$. In order to implement a suitable controller, $e^{r}=0$ should be a fixed point of the error system without controllers (i.e., $u^{r}=0$)

(12) $$\begin{array}{c} \begin{array}{c} {\dot{e}}^{r}=h^{r}\left(Z\right)-\delta h^{r}\left(Y\right) \end{array} \end{array}$$

and $e^{i}=0$ should be a fixed point of the error system in absence of controllers (i.e., $u^{i}=0$)

(13) $$\begin{array}{c} \begin{array}{c} {\dot{e}}^{i}=h^{i}\left(Z\right)-\delta h^{i}\left(Y\right). \end{array} \end{array}$$

Thus, one has

$$\begin{array}{c} \begin{array}{c} \left\{\begin{array}{c} h^{r}\left(\delta Y\right)=\delta h^{r}\left(Y\right),\\ h^{i}\left(\delta Y\right)=\delta h^{i}\left(Y\right). \end{array}\right. \end{array} \end{array}$$

Furthermore, the following equality holds

$$\begin{array}{c} \begin{array}{c} h(\delta Y)=\delta h(Y). \end{array} \end{array}$$

Thus, we obtain the conclusion about the existence of the synchronization problem.

**Theorem** **3.** *The existence of synchronization in complex chaotic system (10) iff h(δY)=δh(Y) has solutions for δ.*

**Proof.**  The proof is easily obtained by Theorem 1 in [12], so it is omitted here. □

Using the result of Theorem 3, one gets that the existence of synchronization in the complex Rikitake system (10) is converted to the following equations having solutions for δ,

$$\begin{array}{c} \begin{array}{c} \left\{\begin{array}{c} h_{1}\left(\delta Y\right)-\delta_{1}h_{1}\left(Y\right)=\left(\delta_{2}\delta_{3}-\delta_{1}\right)y_{2}y_{3}=0,\\ h_{2}\left(\delta Y\right)-\delta_{2}h_{2}\left(Y\right)=\left(\delta_{1}\delta_{3}-\delta_{2}\right)y_{1}y_{3}-\alpha \left(\delta_{1}-\delta_{2}\right)y_{1}=0,\\ h_{3}\left(\delta Y\right)-\delta_{3}h_{3}\left(Y\right)=1-\delta_{3}-\frac{1}{2}\left(\delta_{1}\delta_{2}-\delta_{3}\right)\left({\bar{y}}_{1}y_{2}+y_{1}{\bar{y}}_{2}\right)=0, \end{array}\right. \end{array} \end{array}$$

which leads to

$$\begin{array}{c} \begin{array}{c} \left\{\begin{array}{c} \delta_{1}=\delta_{2},\\ \delta_{3}=1,\\ |\delta_{i}|=1,i=1,2. \end{array}\right. \end{array} \end{array}$$

Furthermore, we have the following results:

I $\delta_{1}=\delta_{2}=\delta_{3}=1$, which implies CS in the complex Rikitake system (10) occurs.
II $\delta_{1}=\delta_{2}=-1,\delta_{3}=1$, which implies the coexistence of CS and AS in the complex Rikitake system (10) exists.

### 4.2. CS of the Complex Rikitake System

Now, we consider CS of two complex Rikitake systems (10) and (11). When $\delta_{1}=\delta_{2}=\delta_{3}=1$, the CS error is defined as e=Z−Y. The error system is calculated as

$$\begin{array}{c} \begin{array}{c} \left\{\begin{array}{c} {\dot{e}}_{1}={\dot{z}}_{1}-{\dot{y}}_{1}=-\beta e_{1}+e_{2}e_{3}+y_{3}e_{2}+y_{2}e_{3}+u_{1},\\ {\dot{e}}_{2}={\dot{z}}_{2}-{\dot{y}}_{2}=-\beta e_{2}-\alpha e_{1}+e_{1}e_{3}+y_{3}e_{1}+y_{1}e_{3}+u_{2},\\ {\dot{e}}_{3}={\dot{z}}_{3}-{\dot{y}}_{3}=-\frac{1}{2}\left({\bar{e}}_{1}e_{2}+e_{1}{\bar{e}}_{2}+{\bar{e}}_{1}y_{2}+e_{1}{\bar{y}}_{2}+{\bar{y}}_{1}e_{2}+y_{1}{\bar{e}}_{2}\right)+u_{3}, \end{array}\right. \end{array} \end{array}$$

which can be rewritten as

(14) $$\begin{array}{c} \begin{array}{c} \left\{\begin{array}{c} {\dot{e}}_{1}^{r}=-\beta e_{1}^{r}+e_{2}^{r}e_{3}+y_{3}e_{2}^{r}+y_{2}^{r}e_{3}+u_{1}^{r},\\ {\dot{e}}_{1}^{i}=-\beta e_{1}^{i}+e_{2}^{i}e_{3}+y_{3}e_{2}^{i}+y_{2}^{i}e_{3}+u_{1}^{i},\\ {\dot{e}}_{2}^{r}=-\beta e_{2}^{r}-\alpha e_{1}^{r}+e_{1}^{r}e_{3}+y_{3}e_{1}^{r}+y_{1}^{r}e_{3}+u_{2}^{r},\\ {\dot{e}}_{2}^{i}=-\beta e_{2}^{i}-\alpha e_{1}^{i}+e_{1}^{i}e_{3}+y_{3}e_{1}^{i}+y_{1}^{i}e_{3}+u_{2}^{i},\\ {\dot{e}}_{3}=-\left(e_{1}^{r}e_{2}^{r}+e_{1}^{i}e_{2}^{i}+e_{1}^{r}y_{2}^{r}+e_{1}^{i}y_{2}^{i}+y_{1}^{r}e_{2}^{r}+y_{1}^{i}e_{2}^{i}\right)+u_{3}, \end{array}\right. \end{array} \end{array}$$

where $\tilde{u}={\left(u_{1}^{r},u_{1}^{i},u_{2}^{r},u_{2}^{i},u_{3}\right)}^{T}$ is a real controller to be designed. Thus, on the basis of Lemma 1, one has the following result.

**Theorem** **4.** *Two identical complex Rikitake systems (10) and (11) can realize CS via the following adaptive controller*

(15) $$\begin{array}{c} \begin{array}{c} \left\{\begin{array}{c} \tilde{u}={\left(0,0,k_{1}e_{2}^{r},k_{1}e_{2}^{i},0\right)}^{T},\\ \dot{k}=-\sigma \left({\left(e_{2}^{r}\right)}^{2}+{\left(e_{2}^{i}\right)}^{2}\right), \end{array}\right. \end{array} \end{array}$$

*where σ>0 is an arbitrary real number.*

**Proof.**  Let us consider the uncontrolled error dynamical system (14). It is clear that if $e_{2}^{r}=e_{2}^{i}=0$, then the subsystem of uncontrolled system (14) reads as

$$\begin{array}{c} \begin{array}{c} \left\{\begin{array}{c} {\dot{e}}_{1}^{r}=-\beta e_{1}^{r}+y_{2}^{r}e_{3},\\ {\dot{e}}_{1}^{i}=-\beta e_{1}^{i}+y_{2}^{i}e_{3},\\ {\dot{e}}_{3}=-y_{2}^{r}e_{1}^{r}-y_{2}^{i}e_{1}^{i}, \end{array}\right. \end{array} \end{array}$$

which is GAS. From Lemma 1, system (14) with controller (15) approaches to the zero equilibrium point, i.e., CS of two identical complex Rikitake systems (10) and (11) can be realized by the designed controller (15). □

In the same argument, when $e_{1}^{r}=e_{1}^{i}=0$, the subsystem of system (14) in absence of controller is presented as

$$\begin{array}{c} \begin{array}{c} \left\{\begin{array}{c} {\dot{e}}_{2}^{r}=-\beta e_{2}^{r}+y_{1}^{r}e_{3},\\ {\dot{e}}_{2}^{i}=-\beta e_{2}^{i}+y_{1}^{i}e_{3},\\ {\dot{e}}_{3}=-y_{1}^{r}e_{2}^{r}-y_{1}^{i}e_{2}^{i}, \end{array}\right. \end{array} \end{array}$$

which is GAS. Thus, the following result is deduced.

**Theorem** **5.** *Two identical complex Rikitake systems (10) and (11) can realize CS by designing adaptive controller*

(16) $$\begin{array}{c} \begin{array}{c} \left\{\begin{array}{c} \tilde{u}={\left(ke_{1}^{r},ke_{1}^{i},0,0,0\right)}^{T},\\ \dot{k}=-\sigma \left({\left(e_{1}^{r}\right)}^{2}+{\left(e_{1}^{i}\right)}^{2}\right), \end{array}\right. \end{array} \end{array}$$

*where σ>0 is an arbitrary real number.*

**Figure 7:**
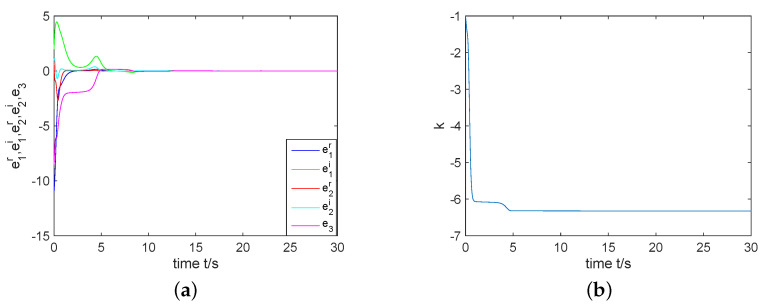
(**a**) CS error system is regulated to the zero equilibrium point; (**b**) *k* approaches to a negative constant.

**Figure 8:**
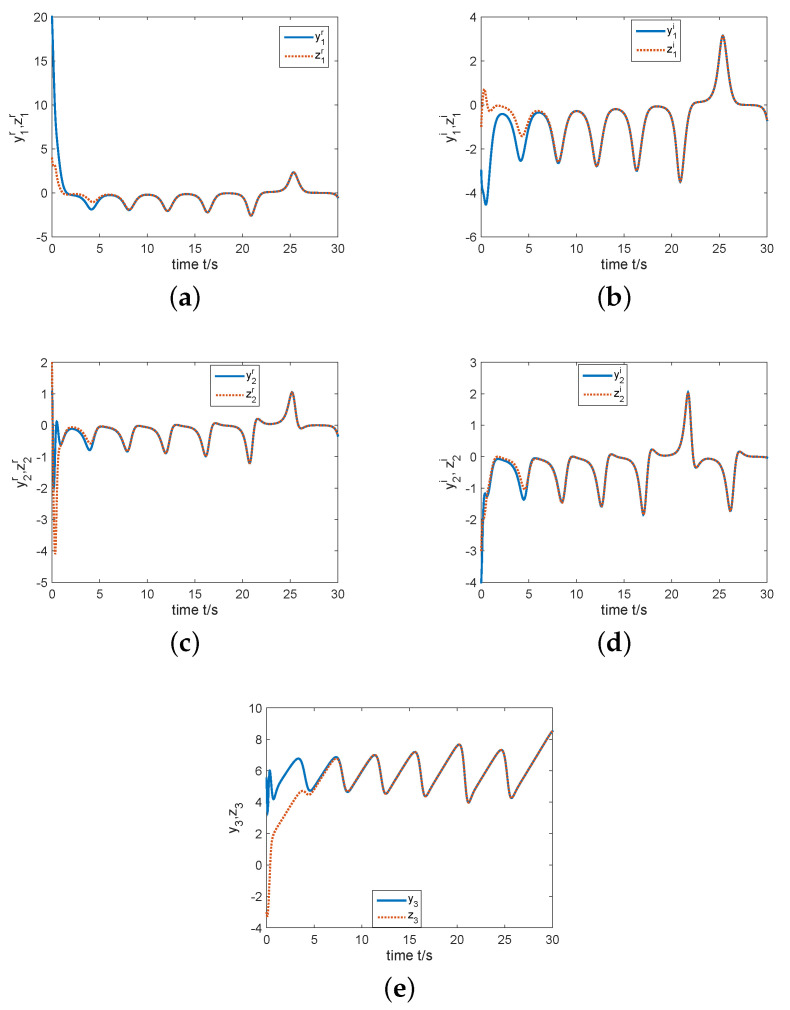
State variables of the complex Rikitake systems (10) and (11) varying time. (**a**) Trajectories of $y_{1}^{r}$ and $z_{1}^{r}$; (**b**) Trajectories of $y_{1}^{i}$ and $z_{1}^{i}$; (**c**) Trajectories of $y_{2}^{r}$ and $z_{2}^{r}$; (**d**) Trajectories of $y_{2}^{i}$ and $z_{2}^{i}$; (**e**) Trajectories of $y_{3}$ and $z_{3}$.

In the next part, by giving the initial conditions as $y\left(0\right)={\left(15-3j,1-4j,5.5\right)}^{T}$, $z\left(0\right)={\left(4-j,2-3j,-0.3\right)}^{T}$, k(0)=−1, σ=2, and constructing controller (15), we have simulation results which are shown by the following Figure 7 and Figure 8. Figure 7a displays that the errors $e_{1}^{r}$, $e_{1}^{i}$, $e_{2}^{r}$, $e_{2}^{i}$ and $e_{3}$ can been regulated to the zero equilibrium point. Figure 8 depicts that state variables of system (11) are completely synchronized with state variables of system (10). That is, two identical complex Rikitake systems realize CS.

### 4.3. The Coexistence of CS and AS in the Complex Rikitake System

When $\delta_{1}=\delta_{2}=-1$, $\delta_{3}=1$, AS error is denoted as $E_{1}=z_{1}+y_{1}$ and $E_{2}=z_{2}+y_{2}$, CS error is denoted as $e_{3}=z_{3}-y_{3}$. It is easy to obtain the following error dynamical system

$$\begin{array}{c} \begin{array}{c} \left\{\begin{array}{c} {\dot{E}}_{1}={\dot{z}}_{1}+{\dot{y}}_{1}=-\beta E_{1}+E_{2}e_{3}+y_{3}E_{2}-y_{2}e_{3}+u_{1},\\ {\dot{E}}_{2}={\dot{z}}_{2}+{\dot{y}}_{2}=-\beta E_{2}-\alpha E_{1}+E_{1}e_{3}+y_{3}E_{1}-y_{1}e_{3}+u_{2},\\ {\dot{e}}_{3}={\dot{z}}_{3}-{\dot{y}}_{3}=-\frac{1}{2}\left({\bar{E}}_{1}E_{2}+E_{1}{\bar{E}}_{2}-{\bar{E}}_{1}y_{2}-E_{1}{\bar{y}}_{2}-{\bar{y}}_{1}E_{2}-y_{1}{\bar{E}}_{2}\right)+u_{3}, \end{array}\right. \end{array} \end{array}$$

which turns into

(17) $$\begin{array}{c} \begin{array}{c} \left\{\begin{array}{c} {\dot{E}}_{1}^{r}=-\beta E_{1}^{r}+E_{2}^{r}e_{3}+y_{3}E_{2}^{r}-y_{2}^{r}e_{3}+u_{1}^{r},\\ {\dot{E}}_{1}^{i}=-\beta E_{1}^{i}+E_{2}^{i}e_{3}+y_{3}E_{2}^{i}-y_{2}^{i}e_{3}+u_{1}^{i},\\ {\dot{E}}_{2}^{r}=-\beta E_{2}^{r}-\alpha E_{1}^{r}+E_{1}^{r}e_{3}+y_{3}E_{1}^{r}-y_{1}^{r}e_{3}+u_{2}^{r},\\ {\dot{E}}_{2}^{i}=-\beta E_{2}^{i}-\alpha E_{1}^{i}+E_{1}^{i}e_{3}+y_{3}E_{1}^{i}-y_{1}^{i}e_{3}+u_{2}^{i},\\ {\dot{e}}_{3}=-E_{1}^{r}E_{2}^{r}-E_{1}^{i}E_{2}^{i}+E_{1}^{r}y_{2}^{r}+E_{1}^{i}y_{2}^{i}+y_{1}^{r}E_{2}^{r}+y_{1}^{i}E_{2}^{i}+u_{3}. \end{array}\right. \end{array} \end{array}$$

**Theorem** **6.** *Two identical complex Rikitake systems (10) and (11) can achieve the coexistence of CS and AS by virtue of the following adaptive controller*

(18) $$\begin{array}{c} \begin{array}{c} \left\{\begin{array}{c} \tilde{u}={\left(0,0,kE_{2}^{r},kE_{2}^{i},0\right)}^{T},\\ \dot{k}=-\sigma \left({\left(E_{2}^{r}\right)}^{2}+{\left(E_{2}^{i}\right)}^{2}\right), \end{array}\right. \end{array} \end{array}$$

*where σ>0 is an arbitrary real number.*

**Proof.**  Let us consider the system (17) in absence of controller. Obviously, when $E_{2}^{r}=E_{2}^{i}=0$, the subsystem of system (17) without controller can be converted to

(19) $$\begin{array}{c} \begin{array}{c} \left\{\begin{array}{c} {\dot{E}}_{1}^{r}=-\beta E_{1}^{r}-y_{2}^{r}e_{3},\\ {\dot{E}}_{1}^{i}=-\beta E_{1}^{i}-y_{2}^{i}e_{3},\\ {\dot{e}}_{3}=y_{2}^{r}E_{1}^{r}+y_{2}^{i}E_{1}^{i}, \end{array}\right. \end{array} \end{array}$$

which is GAS. From Lemma 1, system (17) can be governed at the origin by controller (18). That is to say, the coexistence of CS and AS in two identical complex Rikitake systems (10) and (11) can be realized by adaptive controller (18). □

Similarly, when $E_{1}^{r}=E_{1}^{i}=0$, the subsystem of system (17) without controller is described by

$$\begin{array}{c} \begin{array}{c} \left\{\begin{array}{c} {\dot{E}}_{2}^{r}=-\beta E_{2}^{r}-y_{1}^{r}e_{3},\\ {\dot{E}}_{2}^{i}=-\beta E_{2}^{i}-y_{1}^{i}e_{3},\\ {\dot{e}}_{3}=y_{1}^{r}E_{2}^{r}+y_{1}^{i}E_{2}^{i}, \end{array}\right. \end{array} \end{array}$$

which is GAS. Thus, by means of Lemma 1, we obtain another result.

**Theorem** **7.** *Two identical complex Rikitake systems (10) and (11) can realize the coexistence of CS and AS by designing the following controller*

(20) $$\begin{array}{c} \begin{array}{c} \left\{\begin{array}{c} \tilde{u}={\left(kE_{1}^{r},kE_{1}^{i},0,0,0\right)}^{T},\\ \dot{k}=-\sigma \left({\left(E_{1}^{r}\right)}^{2}+{\left(E_{1}^{i}\right)}^{2}\right), \end{array}\right. \end{array} \end{array}$$

*where σ>0 is an arbitrary real number.*

**Figure 9:**
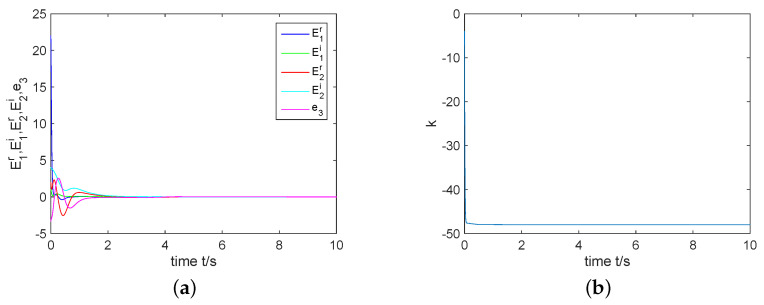
(**a**) The synchronization error system is regulated to the zero equilibrium point; (**b**) *k* is estimated to a negative constant.

**Figure 10:**
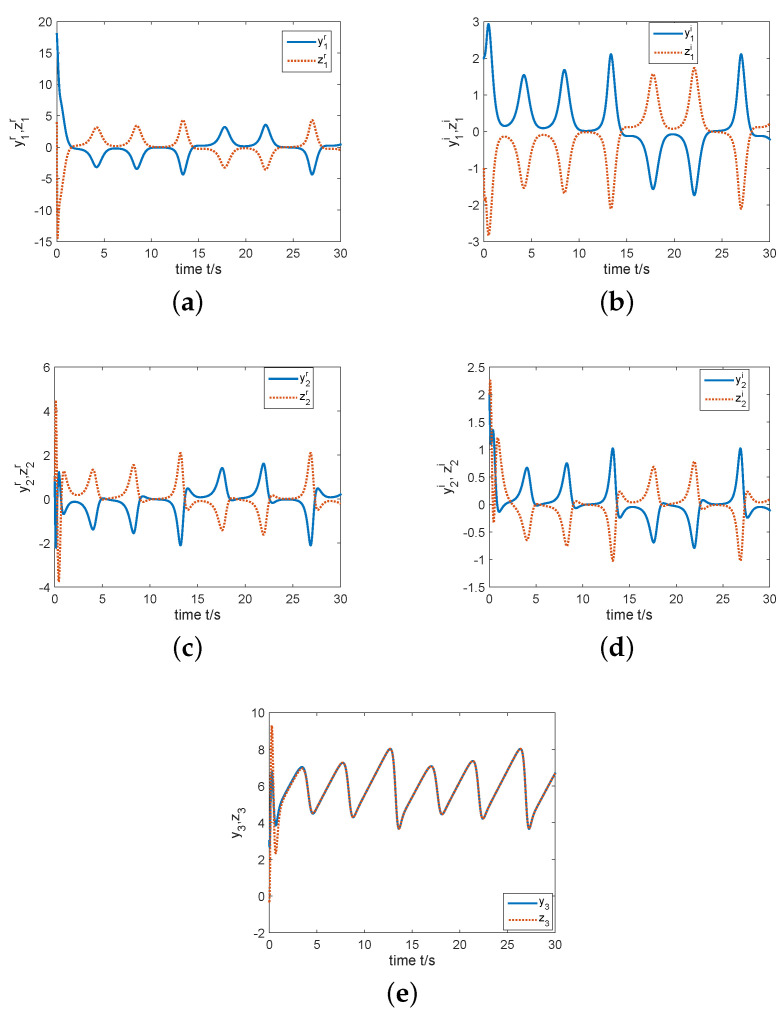
State variables of two identical complex Rikitake systems (10) and (11) varying time. (**a**) Trajectories of $y_{1}^{r}$ and $z_{1}^{r}$; (**b**) Trajectories of $y_{1}^{i}$ and $z_{1}^{i}$; (**c**) Trajectories of $y_{2}^{r}$ and $z_{2}^{r}$; (**d**) Trajectories of $y_{2}^{i}$ and $z_{2}^{i}$; (**e**) Trajectories of $y_{3}$ and $z_{3}$.

For numerical simulations, fix the initial values as $y\left(0\right)={\left(18+2j,1+2j,3\right)}^{T}$ and $z\left(0\right)={\left(4-j,1+2j,-0.3\right)}^{T}$. By constructing controller (20) with k(0)=−4 and σ=5, we can obtain the simulation results, see Figure 9 and Figure 10. As one can see from Figure 9 the errors $E_{1}^{r}$, $E_{1}^{i}$, $E_{2}^{r}$, $E_{2}^{i}$ and $e_{3}$ can be regulated to the zero equilibrium point. Figure 10 describes the change of state variables of systems (10) and (11). It is easy to see that $z_{1}^{r}$, $z_{1}^{i}$, $z_{2}^{r}$ and $z_{2}^{i}$ of system (11) anti-synchronize $y_{1}^{r}$, $y_{1}^{i}$, $y_{2}^{r}$ and $y_{2}^{i}$ of system (10) respectively, while $z_{3}$ of system (11) synchronizes completely with $y_{3}$ of system (10). Therefore, the coexistence of CS and AS in two identical complex Rikitake systems can be realized.

## 5. Conclusions

This paper centers on control and synchronization of a new complex chaotic system. Firstly, we propose a complex Rikitake system and investigate its dynamical behavior. Then, by means of feedback control, we design controllers to regulate the complex Rikitake system to any equilibrium points. Thus, we not only prove the existence of synchronization in the complex Rikitake system but also construct adaptive controllers to realize two types of synchronization schemes, such as CS and the coexistence of CS and AS. It is notable that the presented scheme is a single and linear feedback controller and it is easy to implement in engineering. Therefore, the control method will be widely applied in practice in the future.
